# Impact of emergency department probiotic treatment of pediatric gastroenteritis: study protocol for the PROGUT (Probiotic Regimen for Outpatient Gastroenteritis Utility of Treatment) randomized controlled trial

**DOI:** 10.1186/1745-6215-15-170

**Published:** 2014-05-14

**Authors:** Stephen B Freedman, Sarah Williamson-Urquhart, Suzanne Schuh, Philip M Sherman, Ken J Farion, Serge Gouin, Andrew R Willan, Ron Goeree, David W Johnson, Karen Black, David Schnadower, Marc H Gorelick

**Affiliations:** 1 Sections of Paediatric Emergency Medicine and Gastroenterology, Alberta Children’s Hospital, Alberta Children’s Hospital Research Institute, University of Calgary, 2888 Shaganappi Trail NW, Calgary, AB T3B 6A8, Canada; 2 Paediatric Emergency Research Team, Alberta Children’s Hospital, University of Calgary, 2888 Shaganappi Trail NW, Calgary, AB T3B 6A8, Canada; 3 Division of Paediatric Emergency Medicine, The Hospital for Sick Children, University of Toronto, 555 University Avenue, Toronto, ON M5G 1X8, Canada; 4 Division of Gastroenterology, Hepatology and Nutrition, The Hospital for Sick Children, University of Toronto, 555 University Avenue, Toronto, ON M5G 1X8, Canada; 5 Departments of Pediatrics and Emergency Medicine, University of Ottawa, 401 Smyth Road, Ottawa, ON K1H8L1, Canada; 6 Emergency Department, Children’s Hospital of Eastern Ontario, 401 Smyth Road, Ottawa, ON K1H8L1, Canada; 7 Division of Paediatric Emergency Medicine, Department of Pediatrics, Centre Hospitalier Universitaire Ste-Justine, Université de Montréal, 3175 Côte-Sainte-Catherine, Montréal, Québec H3T1C5, Canada; 8 Program in Child Health Evaluative Sciences, SickKids Research Institute, Dalla Lana School of Public Health, University of Toronto, 555 University Avenue, Toronto, ON M5G 1X8, Canada; 9 Division of Pediatric Emergency Medicine, Washington University School of Medicine, 660 S. Euclid Avenue, Campus Box 8116, St. Louis 63110MO, USA; 10 PATH Research Institute, St. Joseph's Healthcare Hamilton, Department of Clinical Epidemiology and Biostatistics, McMaster University, 25 Main Street West, Suite 2000, Hamilton, ON L8P1H1, Canada; 11 Departments of Pediatrics and Physiology and Pharmacology, Faculty of Medicine, Alberta Children’s Hospital Research Institute, University of Calgary, 2888 Shaganappi Trail NW, Calgary, AB T3B 6A8, Canada; 12 Division of Pediatric Emergency Medicine, University of British Columbia, BC Children’s Hospital, 4480 Oak St, Vancouver, BC V6H3N1, Canada; 13 Department of Pediatrics, Medical College of Wisconsin, Children’s Hospital of Wisconsin, 8915 W. Connell Ave., P.O. Box 1997, Milwaukee, WI 53226, USA

**Keywords:** Probiotics, Emergencies, Pediatrics, Gastroenteritis, Randomized controlled trial, Adverse effects, Immunoglobulin A

## Abstract

**Background:**

The burden of acute gastroenteritis on children and their families continues to be enormous. Probiotics, defined as viable microbial preparations that have a beneficial effect on the health of the host, represent a rapidly expanding field. Although clinical trials in children with gastroenteritis have been performed, most have significant flaws, and guidelines do not consistently endorse their use.

**Methods/Design:**

PROGUT is a randomized, placebo-controlled, double-blind, five-center, Canadian, emergency department trial. Children aged 3 months to 48 months who present between November 2013 and June 2017 with <72 hours of gastroenteritis symptoms will be assessed for eligibility. A total of 886 children will be randomized (1:1 allocation via an internet based, third party, randomization service) to receive 5 days of a combination probiotic agent (*Lactobacillus rhamnosus* and *L. helveticus*) or placebo. All participants, caregivers, and outcome assessors will be blinded to group assignment. The study includes three key outcomes: 1) clinical - the development of moderate to severe disease following an emergency department (ED) evaluation that employs a validated clinical score (Modified Vesikari Scale); 2) safety - side effect; and 3) mechanism - fecal secretory immunoglobulin A levels.

**Discussion:**

Definitive data are lacking to guide the clinical use of probiotics in children with acute gastroenteritis. Hence, probiotics are rarely prescribed by North American physicians. However, the following current trends obligate an urgent assessment: 1) probiotics are sold as food supplements, and manufacturers can encourage their use while their relevance has yet to be established; 2) North American and European government agencies remain concerned about their value and safety; 3) some institutions are now recommending the routine use of probiotics; and 4) parents of affected children are often providing probiotics. With probiotic consumption increasing in the absence of solid evidence, there is a need to conduct this definitive trial to overcome the limitations of prior work in this field.

**Trial registration:**

ClinicalTrials.gov: NCT01853124; first registered 9 May 2013.

## Background

In the United States each year, there are approximately 179 million episodes of acute gastroenteritis (AGE) [1,2]. Children often suffer from prolonged [3] and severe illness; among hospitalized children, 19% have clinical sepsis, 7% seizures, and 4% require intensive care unit admission [4]. Apart from supportive care, healthcare providers have little to offer to relieve suffering [5].

Probiotics, which are viable microbial preparations that have a beneficial effect on the health of the host, [6] represent a rapidly expanding field. Meta-analyses on their use in children with AGE [7-11] are encouraging; however, they question the relevance of the outcomes evaluated [7,11,12] and advocate for large randomized clinical trials (RCT) [13] in ambulatory pediatric populations [7]. Consequently, in North America, probiotics are rarely used [14-19]. However, current trends obligate an urgent assessment for several reasons. First, probiotics are sold as food supplements, and manufacturers encourage their use through campaigns, making health claims that are not be supported by rigorous research [20-23]. At stake is the $33 billion/year worldwide probiotic market, which is growing at 13% annually [24]. Second, government agencies remain concerned about the value and safety of probiotics [25-27]. Third, some institutions are recommending the routine use of probiotics [28]. Finally, parents are often providing probiotics [16], and consumption is increasing in the absence of evidence.

Prior research suffers from the following shortcomings:

1. Outcome measures used to date have limited clinical meaning. That is, studies have focused on individual symptoms, without consideration of the full picture of the illness [29]. Thus, the significance of the conclusions is questioned [12,30].

2. Quality of studies is inadequate. Most are small, single-center studies [11] that have been conducted by pharmaceutical companies [7]. Many negative studies remain unpublished [31]. Design issues are a concern; only 16% of studies adequately reported the four key methodological assessment parameters [11].

3. Inadequate data are available from research in the relevant patient population. Though 95% or more of children are treated as outpatients [32], only a handful of small studies have focused on outpatients [12]. Hospitalized children are more likely to benefit from probiotics [9,11,33]. Only a single ED study has been performed. In this study, 129 children received a probiotic or placebo agent, and the authors found statistically insignificant trends towards a reduction in stool frequency and duration among those administered a probiotic [34].

4. Knowledge about the *in vivo* mechanism of action in AGE is limited [35,36].

In light of the above considerations, we have obtained funding from the Canadian Institutes for Health Research (CIHR) to conduct the PROGUT (Probiotic Regimen for Outpatient Gastroenteritis Utility of Treatment) trial employing a combination product, Lacidofil™, which contains *Lactobacillus rhamnosus R0011* (95%) and *L. helveticus R0052* (5%).

## Methods/Design

### Hypotheses

In children aged 3 to 48 months of age who present with less than 72 hours of AGE-like symptoms to an ED, the administration of a probiotic agent when compared with a placebo:

1. will result in a significantly lower proportion of children developing moderate to severe disease over the subsequent 2 weeks.

2. will not be associated with a significantly greater occurrence of minor side effects.

3. will be associated with a greater increase in secretory IgA (sIgA).

### Study questions

#### Clinical efficacy

Clinical efficacy will be determined as follows:

1. For previously healthy children, ages 3 to 48 months, who present with less than 72 hours of AGE-like symptoms to an ED, is the probability of developing moderate to severe disease (Modified Vesikari Scale (MVS) score ≥9) following ED evaluation, significantly different in those who receive Lacidofil compared to those who receive placebo?

2. Is there a difference in the (a) mean duration of diarrhea or (b) mean duration of vomiting?

3. Is there a difference in the probability of requiring an unscheduled healthcare provider visit?

#### Side effect profile

In this group of patients, is the proportion that experiences a side effect (for example, bloating, fever, abdominal distention, or rash) significantly different in those who receive Lacidofil compared to placebo?

#### Mechanism of action

In this group of patients, are fecal sIgA levels 5 days and 4 weeks after the initiation of treatment higher in those who receive Lacidofil compared to those who receive placebo?

### Study design

This a randomized, placebo-controlled, double-blind, five-center, ED-based trial. A total of 886 children will be randomized to receive 5 days of Lacidofil [8 × 10^9^ colony forming units (CFU)/day] or placebo.

### Ethics approval

Site-specific approval has been granted by the local ethics committees at the following study sites: University of Calgary, The Hospital for Sick Children (HSC), Children’s Hospital of Eastern Ontario, Centre Hospitalier Universitaire Ste-Justine, and the IWK Health Centre. A Notice of Authorization has been granted by Health Canada’s Health Product and Food Branch, Biologics and Genetic Therapies Directorate (file #: 9427 - U0206 - 77C).

### Study population

The diagnosis of gastroenteritis is at the discretion of the ED supervising physician and symptoms may or may not include vomiting. Alternative terminologies that reflect as similar diagnosis are acceptable (for example, viral illness, diarrhea, vomiting, upper respiratory infection, post-infectious gastroenteritis, antibiotic associated diarrhea, toddler’s diarrhea, viral infection, enteritis, viremia, fever, and bronchiolitis).

#### Inclusion criteria

All of the following must be met for inclusion:

1. ≥ 3 watery stools in a 24-hour period [37]

2. duration of vomiting or diarrhea <72 hours

3. age 3 to <48 months

#### Exclusion criteria

Exclusion criteria include:

1. presence of an indwelling vascular access line or structural heart disease [38].

2. taking immunosuppressive therapy, or known history of immunodeficiency [39].

3. hematochezia in the preceding 72 hours, underlying significant chronic gastrointestinal problem or inflammatory bowel disease. This does not including constipation, gastroesophageal reflux or chronic pain.

4. family member with an indwelling vascular access line, on immunosuppressive therapy, or with a known immunodeficiency. This does not include use of short course oral or inhaled steroids.

5. bilious vomiting.

6. supplemental probiotic use in the preceding 2 weeks. The consumption of foods containing probiotics will not result in exclusion.

7. previously enrolled in this trial.

8. daily communication will not be possible while symptomatic.

9. allergy to soy.

10. pre-existing, or known, pancreatic dysfunction or insufficiency [40].

11. oral or gastrointestinal surgery within the preceding 7 days.

### Intervention

Informed consent will be obtained from each participant’s legally authorized guardian prior to enrollment into the study. Once consent is provided, the first dose will be administered in the ED. The sachet’s contents will be sprinkled into 30 mL of oral rehydration solution (ORS). Caregivers will receive instructions on study drug administration, completion of study forms, what and how much fluid to drink, criteria for seeing a health care practitioner or returning to the ED [see Additional file 1] and standardized AGE discharge instructions.

All patients will take one sachet every 12 hours for 5 days, at meal time, even once symptoms have resolved. The dose will be repeated once should vomiting occur within 15 minutes of medication administration. Children who are hospitalized will continue as per study protocol.

### Investigational agents

We have obtained independent analyses to confirm the viable CFU count and microbe identity [see Additional files 2 and 3]. Lacidofil data indicate that a dose of 3 to 6 × 10^9^ CFU/day is effective [41]. A recent pilot trial, which employed low (4 × 10^9^ CFU/day) and high (8 × 10^9^ CFU/day) dose arms, found no side effects with either dose. However, a positive association is postulated to exist between the probiotic dose and clinical benefits [7], with most positive studies employing doses ≥6 × 10^9^ CFU/day [9]. Thus, a dose of 8 × 10^9^ CFU/day is being employed for the current study. The duration of therapy has been selected based on the best available evidence, the recommendations of experts in the field, previous studies, and the typical duration of most episodes of AGE [42].

### Stool sample testing

Stool samples from all participants will be tested for bacteria and viruses. For children who do not provide a stool specimen prior to discharge, rectal swabs will be performed. For patients enrolled at HSC and Alberta Children’s Hospital (ACH), additional stool samples will be collected on Days 5 and 28 for sIgA testing.

### Randomization

#### Sequence generation

We used http://www.randomize.net to produce the study randomization lists stratified by site. The lists were sent to the central pharmacy at ACH where an independent research pharmacist prepared consecutively numbered kits according to the randomization schedule. The kits were couriered directly to the sites using insulated shipment containers and temperature monitors.

#### Allocation concealment

The site http://www.randomize.net uses industry standard security to send data over the internet. Randomization employed random blocks of 4 and 6 with a 1:1 allocation ratio.

#### Implementation

A log of all screened patients is being maintained. If consent is obtained, study staff collects baseline demographic clinical variables and complete the data collection forms. Study staff then log into http://www.randomize.net to randomize the patient.

### Bias

The probiotic and placebo powders are identical in appearance, taste, texture and smell. Participants, families, healthcare providers, data collectors, outcome adjudicators, and data analysts are blinded. Co-interventions and other sources of confounding are being recorded. The trial has been registered at http://www.clinicaltrials.gov (NCT01853124).

### Concomitant medications

The concomitant administration of antibiotics is permitted as probiotics remain effective when given concomitantly with antibiotics [43]. Their use is at the discretion of the child’s treating physician. Similarly, antipyretics, anti-emetics, and other medications may be administered. ORS will be provided during the ED visit. Discharge instructions will be provided.

### Outcomes

#### Primary outcome (clinical)

The primary outcome is the development of moderate to severe disease in the 2 weeks after the index ED visit as measured by the MVS (Table 1) [44]. The original 20 point Vesikari Score, which has been employed as a dichotomous variable in many clinical studies [45-53], correlates with other meaningful measures such as caregiver anxiety, helplessness, stress, [54] parental worry, behavioral changes, and impact on the parents’ daily activities and distress [55]. However, percent dehydration, an element of the original score, is challenging to quantify due to difficulties in collecting follow-up weights, determining when rehydration has occurred, and the variation related to timing of voiding, stooling, eating, and drinking. Thus, this element is omitted or incorrectly assigned in many studies. The modified score includes an important and easy to obtain outcome that reflects global disease severity - need for unscheduled healthcare provider visits within 2 weeks of the index visit [44]. This is supported by evidence that the utilization of professional medical care correlates with disease severity [54].

**Table 1:** Modified Vesikari Scale

**Points**	**0**	**1**	**2**	**3**
**Diarrhea duration**	0	1 to 96 hours	97 to 120 hours	≥121 hours
**Maximum number of diarrheal stools/24-hour period**	0	1 to 3	4 to 5	≥6
**Vomiting duration (d)**	0	1 to 24 hours	25 to 48 hours	≥49 hours
**Maximum number of vomiting episodes/24-hour period**	0	1	2 to 4	≥5
**Maximum recorded fever**	<37.0˚C R	37.1 to 38.4˚C R	38.5 to 38.9˚C R	≥39.0˚C R
**Future healthcare visit**	0%	-	Primary care	Emergency department
**Treatment**	None	Rehydration	Hospitalization	-

R, rectal.

Follow-up will occur daily until both the diarrhea and vomiting have resolved. On Day 14 each variable is assigned a score for the entire study period (Time 0 to Day 14); each patient gets a single total score for the study. Variables are scored based on the worst 24-hour period or on the total duration of symptoms or are based on the occurrence of an outcome.

Regardless of the score assigned at Time 0 (that is, the pre-enrollment score), everyone reverts to a score of 0 at enrollment. The pre-enrollment score will serve as a covariate in a secondary analysis of the primary outcome and will be employed for subanalysis purposes. The primary outcome will only include symptoms and outcomes that occur following the ED visit and will not be directly impacted by the pre-enrollment score.

With the original score, severe disease was defined as ≥11 [45,46,51,52,56-58] and moderate as ≥9 [59]. In the derivation study, [44] construct validity was proven by using scores of ≥9 to define moderate and ≥11 to define severe disease. These cut-points were associated with significant increases in other measures of disease severity (for example, daycare (*P* = 0.01) and work absenteeism (*P* = 0.002)) [44].

#### Secondary outcomes

Clinical secondary outcomes are:

1. duration of diarrhea, which is time from treatment initiation until the appearance of the last watery stool [60-62].

2. duration of vomiting. Recovery will be evaluated in children who vomit ≥3 times over the 24 hours prior to the ED visit, and duration is defined as ‘time from treatment initiation until last vomiting episode’.

3. return visits for unscheduled care to a healthcare provider related to vomiting, diarrhea, dehydration, fever, or fluid refusal, within two weeks. Scheduled visits will not be included.

4. work and daycare absenteeism.

#### Safety

To determine if short course probiotic administration to young children with AGE is associated with an increase in minor side effects, groups will be compared regarding the development of any side effects with particular attention paid to bloating or abdominal distention (grouped for analysis), duration of fever, and buttock rash.

#### Mechanism

To determine if probiotic administration increases fecal sIgA levels in children with AGE, the first stool sample produced following enrollment will be collected along with samples on Days 5 and 28. We will determine if fecal sIgA levels are greater among children treated with a probiotic agent compared with placebo. Levels will be correlated with clinical findings.

### Data retrieval

1. Daily Telephone/Electronic Survey Communication: At the index visit, caregivers will be asked their preferred method of communication - electronic versus telephone. Caregivers will be contacted daily by the identified method until both the diarrhea and vomiting have resolved. A standardized collection form will be employed. Detailed questioning will follow positive responses. Compliance will be assessed on Day 5. To maximize validity, caregivers will be reminded of the importance and method of administering the probiotic/placebo.

2. Chart Review: We will verify data regarding revisits, intravenous hydration, hospitalization, and microbiology testing using each center’s medical record database.

3. Database Reviews: Provincial databases and Canadian Institute for Health Information databases will be employed to verify future health care provider use.

### Health service research issues

An economic evaluation will be conducted alongside the RCT. The incremental cost effectiveness will be determined by assessing resources and costs associated with the treatment of AGE for children who receive the current standard of care compared to those who receive a probiotic.

### Sample size estimates

#### Clinical

The sample size estimate was based on the assessment of the between-group difference in proportions of children with a post-randomization score ≥9 on the MVS. The adoption of probiotic use can be recommended if the proportion of the primary outcome is significantly lower among those who receive the probiotic medication. Calculations were based on a two-sided α of 0.05 and power of 0.90. The null hypothesis is *H*_*0*_: *P*_*c*_ - *P*_*I*_ = 0, where *P*_*I*_ and *P*_*C*_ are the outcome probabilities in the intervention and control groups, respectively. The alternative hypothesis is *H*_*A*_*:│P*_*I*_ - *P*_*C*_*│* >0.10. Ten content experts from the United States and Canada were surveyed regarding the minimal clinically important difference; absolute risk differences ranging from 7.5 to 15% were suggested. A conservative estimate of 10% was selected for the primary outcome.

Our estimate for the development of moderate to severe AGE in the controls is based on data collected as part of two evaluations of the MVS in nearly 750 children in U.S. [63] and Canadian EDs. [44] Approximately 25% of eligible children had scores consistent with moderate to severe disease. Employing a baseline probability of 25% in the controls, the required sample size to compare proportions between two groups is 670 [64]. Based on previous work by our group [65-67], we assume a 10% loss to follow-up, 5% drop out, and 2.5% drop in probabilities. Adjustment for O’Brien-Fleming monitoring boundaries requires a further 2% increase. Thus, the final sample size required is 886.

#### Safety

RCTs employing probiotics have not attributed any adverse events to probiotic administration [11]. Given our sample size, a significant difference between groups will be easily detected.

#### Mechanism

A study evaluating the impact of formula supplementation with oligosaccharides found fecal sIgA values of 729 and 377 μg/g in the intervention and control groups, respectively [68]. Assuming a clinically significant difference of 300 μg/g, a standard deviation of 500 μg/g, 80% power and a type I error of 0.05, the required sample size is 45 subjects/group. We will recruit 100 patients to provide multiple stool samples for this phase of the study.

### Statistical analysis

All analyses will be undertaken by the intention-to-treat principle. Adverse events will use the ‘as treated’ principle. Patients who drop out or crossover will be followed and included. All statistical tests will be two-sided. Baseline characteristics will be compared between groups using frequency counts and percentages for discrete variables, and means, medians, standard deviations, and interquartile ranges for continuous variables. Sensitivity analyses will be performed to assess the possibility and consequences of losses to follow-up not occurring at random.

#### Clinical

The proportion of children with moderate to severe disease will be analyzed by utilizing a Mantel-Haenszel test, stratified by clinical center. Significance for the primary outcome measure will be determined using a two-sided 0.05 level. Secondary analyses of the primary outcome will employ logistic regression methods to adjust for covariates that may be imbalanced between groups. We will analyze the MVS as a continuous variable through a stratified Wilcoxon rank-sum test. The overall significance level for statistical tests on the secondary outcomes will be set at 0.05. Holm’s method will be used to adjust for multiple comparisons. The continuous variables of duration of diarrhea and vomiting will be measured in hours and analyzed with a Van Elteren test, stratified by clinical center. Unscheduled healthcare visits will be analyzed using a Mantel-Haenszel test, stratified by clinical center. The tertiary outcomes of the number of days the child is absent from daycare and the days the caregiver is absent from work will be analyzed using an appropriate model with robust estimates for standard errors. Dichotomous outcomes to be evaluated but unlikely to achieve significance include ED revisits, intravenous rehydration, and hospitalization. Additional analyses involving these outcomes will include linear and logistic regression models that adjust for possible effects of baseline characteristics.

#### Safety

The proportions of children experiencing any side effect, as reported by the caregivers, will be compared between groups using the Mantel-Haenszel test, stratified by site. The analysis will evaluate the presence/absence of side effects, as an aggregate outcome variable.

#### Mechanism

To test for a difference in fecal secretory IgA the Wilcoxon rank-sum test will be performed. As this is a mechanistic outcome and the motivation of its study is distinct from other outcomes, the test will be performed at the 0.05 level. Data will be analyzed to determine if fecal secretory IgA levels 5 days and 4 weeks after initiation of treatment are higher among children treated with probiotic than those treated with placebo. Fecal sIgA data will also be analyzed by outcome, comparing levels among those with mild disease to those with moderate to severe disease.

#### Planned subgroup analyses

1. The presence of a MVS ≥9 will be analyzed by (i) age <1 year, (ii) breast-feeding status, (iii) antibiotic usage and (iv) protocol compliance.

2. Duration of vomiting will be analyzed only in those patients who have had ≥3 episodes of vomiting in the 24 hours prior to enrollment.

3. Daycare and work absenteeism will only be analyzed for children who attend daycare and caregivers who work.

4. In children with rotavirus infection, an interaction term will be added between treatment and rotavirus positivity in a logistic regression model. The independent variables in the model will be (i) treatment group, (ii) rotavirus positivity (yes/no) and (iii) the interaction between treatment group and rotavirus positivity.

5. Fecal sIgA levels will be subanalyzed based on the mother’s breast-feeding status.

### Safety

The Data Safety Monitoring Committee (DSMC) will meet after 200 and 500 patients to review enrollment, study procedures, form completion, data quality, loss to follow-up, drop-in rate, and interim safety and efficacy results. The analyses will test the hypothesis that the probability of developing moderate to severe AGE in the probiotic arm is equal to that in the placebo arm. Conservative O’Brien-Fleming monitoring boundaries, implemented using the Lan-DeMets alpha-spending function approach, will be used as guidelines for early stopping for safety or efficacy. Based on trends and adverse events, the DSMC may decide to meet sooner than planned using boundaries adjusted accordingly. Because this trial involves children under the age of 6 months, the DSMC has approved a plan to complete an interim safety analysis on the first 20 subjects enrolled under 6 months of age. All serious adverse events will be reported within 24 hours to the DSMC and based on these reports; the DSMC may decide to conduct a safety analysis before the full 20 subjects have been enrolled in this age group. Otherwise, a blinded analysis will be conducted after the 20 subjects <6 months of age have been enrolled. This data will be unblinded if the DSMC deems it necessary to conduct an unblinded interim safety analysis. The results of this analysis will be communicated to Health Canada at the discretion of the DSMC chair should any concerns be identified. The DSMC consists of a biostatistician (Nick Barrowman, PhD-Ottawa), and two physicians with RCT expertise (Drs. Mark Roback-Minnesota and Terry Klassen (Chair) -Winnipeg).

An adverse event has been defined as any unfavorable or unintended clinical or other occurrence during the study period that may or may not be the result of participation in the research study.

Expected adverse drug reactions/events include the following, which were deemed to be part of the natural history of the underlying disease process:

1. hospitalization

2. future healthcare provider visit, ED return visit

3. IV rehydration

4. abdominal pain, distension

5. vomiting, diarrhea, fever, flatulence

Because expected adverse events are part of the natural history of AGE and diarrheal illness in children, they will not need to be reported as adverse events. This information will be recorded in normal study data collection processes. Any serious adverse event (SAE) that occurs after the first sachet administered will be reported to the Research Ethics Board (REB) and the study subject will be followed until the conclusion of the event. Any serious adverse event to the natural health product will be reported to Health Canada. An SAE has been defined as any of the following:

1. results in death.

2. is life-threatening. This refers to an event in which the patient was at immediate risk of death; it does not refer to an event that might have caused death had it been more severe.

3. results in a persistent or significant disability/incapacity.

4. is medically significant. Important medical events that may not result in death, be life-threatening, or require hospitalization may be considered SAEs when, based upon appropriate medical judgment, may jeopardize the patient and may require medical or surgical intervention to prevent one of the outcomes listed in this definition.

Unblinding should only occur in the event that there is clinical concern regarding the possibility of bacteremia/septicemia or when it is felt by the treating physician that unblinding would alter the clinical care being provided. All patients whose therapy is intentionally unblinded will discontinue the experimental therapy. Approval from the principal investigator or designate will be obtained prior to unblinding. If the principal investigator cannot be reached, unblinding can be performed and the principal investigator informed within 24 hours via email or telephone.

Subjects will be withdrawn from the study if:

1. after enrollment they are determined to meet any of the exclusion criteria.

2. the subject is admitted to an intensive care unit.

3. it is deemed by the treating physician that the child’s health may be jeopardized by continued participation in the study.

4. the patient’s caregivers wish to withdraw their child for whatever reason.

### Trial management

The Clinical Research Informatics Core (CRIC), based at the University of Alberta, will act as a central repository for all study data and they will be responsible for the provision of data collection technology and clinical data management services. Dr. Willan will supervise all data analyses. Dr. Freedman takes overall responsibility for the study. Double data entry will be employed on a random sampling of subjects at various time points throughout the study. The study has a Steering Committee that includes senior clinical research team members (Drs. Gorelick, Schuh, and Johnson), Dr. Sherman (gastroenterologist), Dr. Kuppermann (past-Chair of PECARN), Dr. Dean (Director of the Central Data Management and Coordinating Center for PECARN), and Dr. Plint (Chair of PERC).

## Discussion

It should be noted that a similar study is also being conducted in the United States with funding provided by the National Institutes of Health (NCT01773967). The study will be employing very similar study design; however, a different probiotic agent (*Lactobacillus* GG) will serve as the investigational agent. These parallel studies will provide a unique opportunity to conduct meta-regression analyses, and together they will provide a clear message regarding the use of probiotic products in this study population.

## Trial status

As of 16 April 2014, 152 children have been enrolled at the five study sites.

## Abbreviations

ACH: Alberta Children’s Hospital; AGE: acute gastroenteritis; CFU: colony forming unit; CIHR: Canadian Institutes for Health Research; CRIC: Clinical Research Informatics Core; DSMC: Data Safety Monitoring Committee; ED: emergency department; HSC: Hospital for Sick Children; IgA: immunoglobulin A; MVS: Modified Vesikari Scale; ORS: oral rehydration solution; PROGUT: Probiotic Regimen for Outpatient Gastroenteritis Utility of Treatment; RCT: randomized clinical Trial; REB: Research Ethics Board; SAE: serious adverse event; sIgA: secretory immunoglobulin A.

## Competing interests

Dr. Stephen Freedman discloses having received financial support from Lallemand Health Solutions in 2008 for the conduct of the pilot study that provided safety data, which was employed to determine the dose of Lacidofil employed in the current protocol. Lallemand Health Solutions is supplying the investigational agent and placebo for the current clinical trial. The authors declare no other competing interests.

## Authors’ contributions

SF conceived and designed the study. The study protocol and manuscript were written and critically reviewed by SU, SS, PS, KF, SG, AW, RG, DJ, KB, DS, and MG. All authors read and approved the final manuscript.

## Supplementary Material

Additional file 1 Discharge instruction list provided to study participants (Canada). Click here for file

Additional file 2 Lactobacilli enumeration. Click here for file

Additional file 3 Identification of the bacterial population present using randomly amplified polymorphic DNA technique. Click here for file
