# Supplementary material for: Impact of emergency department probiotic treatment of pediatric gastroenteritis: study protocol for the PROGUT (Probiotic Regimen for Outpatient Gastroenteritis Utility of Treatment) randomized controlled trial
Source: Trials. 2014 May 14;15:170. doi: 10.1186/1745-6215-15-170 (PMC4037747; doi:10.1186/1745-6215-15-170)
Supplement: Additional file 1 — Discharge instruction list provided to study participants (Canada). [file 1745-6215-15-170-S1.doc]

**Additional File 1. Discharge instruction list provided to study participants (Canada).**
