# Supplementary material for: Impact of emergency department probiotic treatment of pediatric gastroenteritis: study protocol for the PROGUT (Probiotic Regimen for Outpatient Gastroenteritis Utility of Treatment) randomized controlled trial
Source: Trials. 2014 May 14;15:170. doi: 10.1186/1745-6215-15-170 (PMC4037747; doi:10.1186/1745-6215-15-170)

**Additional File 3. Identification of the bacterial population present using randomly amplified polymorphic DNA technique.**

**
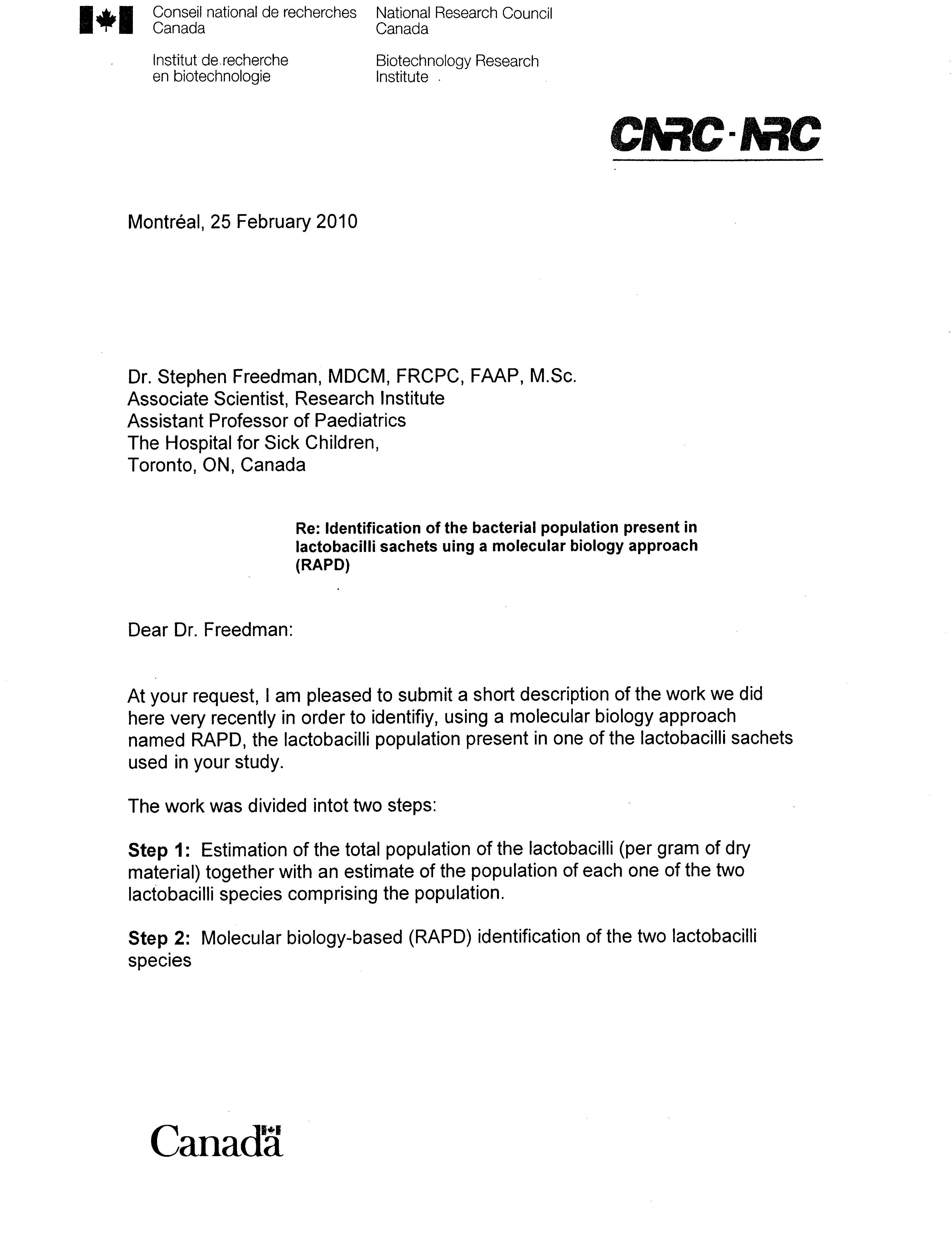
**


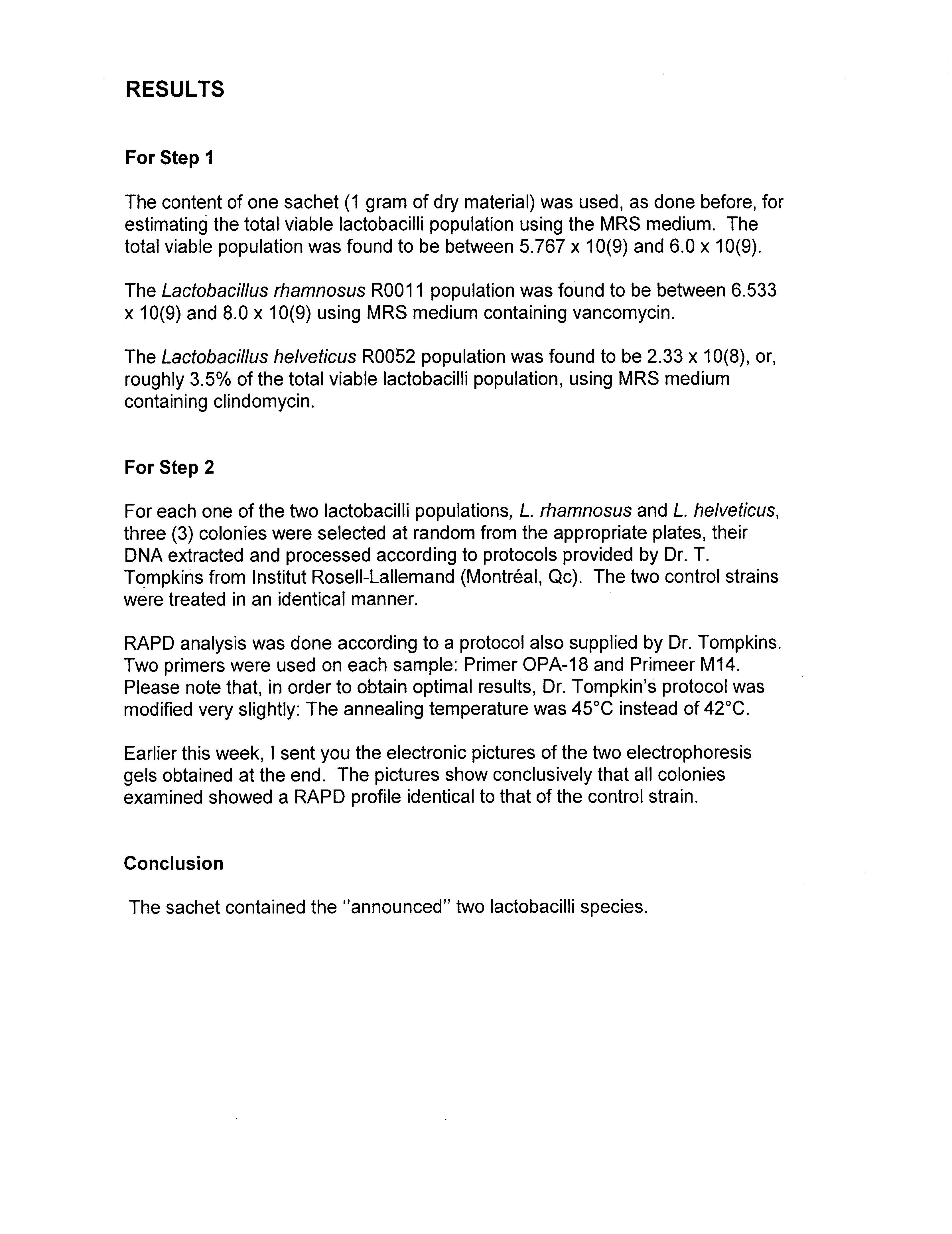


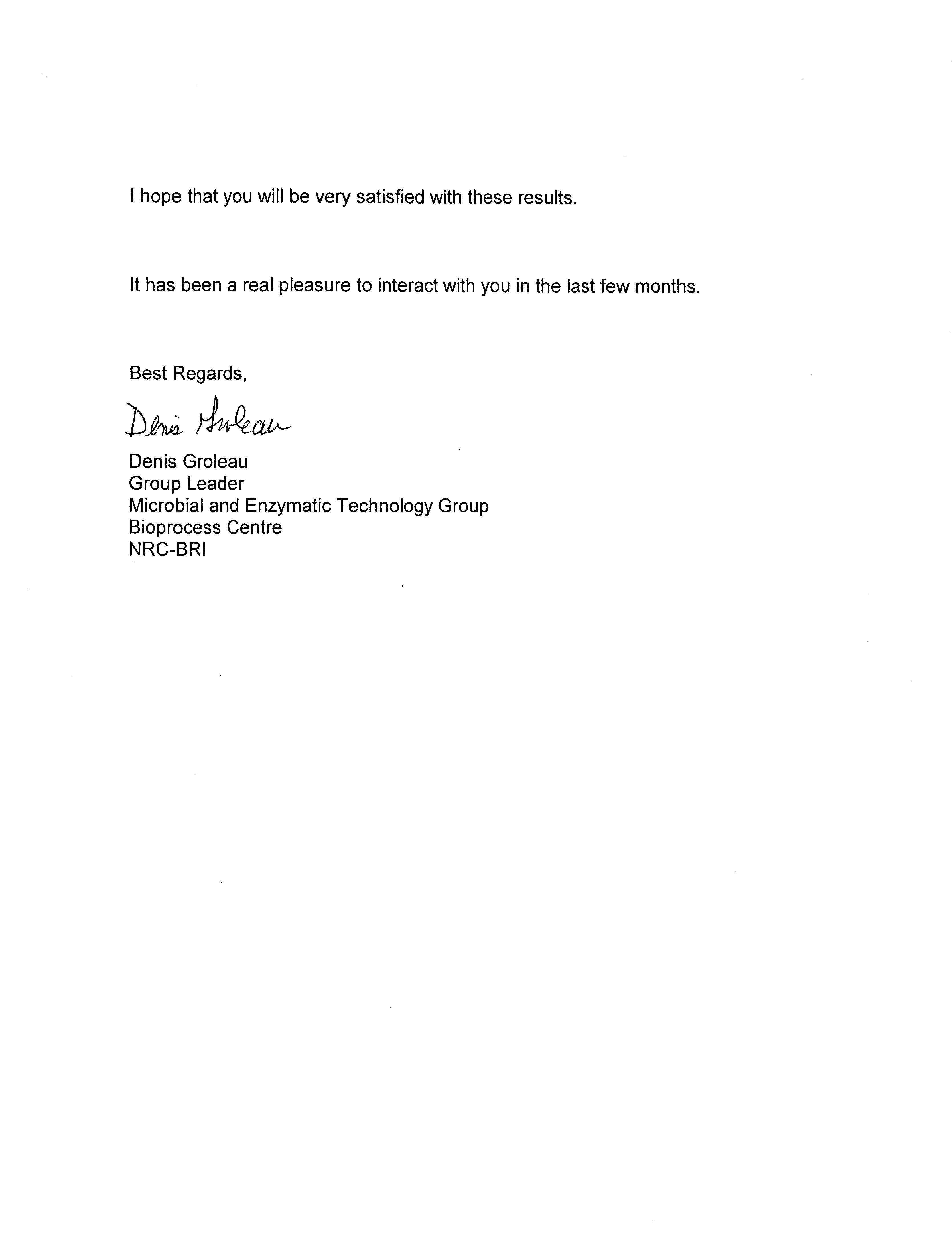

Supplement: Additional file 3 — Identification of the bacterial population present using randomly amplified polymorphic DNA technique. [file 1745-6215-15-170-S3.doc]
